# Equilibrium shape of single-layer hexagonal boron nitride islands on iridium

**DOI:** 10.1038/s41598-019-56000-1

**Published:** 2019-12-20

**Authors:** Marin Petrović, Michael Horn-von Hoegen, Frank-J. Meyer zu Heringdorf

**Affiliations:** 1 Faculty of Physics and CENIDE, University of Duisburg-Essen, Lotharstr. 1, D-47057 Duisburg, Germany; 2 Center of Excellence for Advanced Materials and Sensing Devices, Institute of Physics, Bijenička cesta 46, HR-10000 Zagreb, Croatia

**Keywords:** Surfaces, interfaces and thin films, Synthesis and processing, Two-dimensional materials

## Abstract

Large, high-quality layers of hexagonal boron nitride (hBN) are a prerequisite for further advancement in scientific investigation and technological utilization of this exceptional 2D material. Here we address this demand by investigating chemical vapor deposition synthesis of hBN on an Ir(111) substrate, and focus on the substrate morphology, more specifically mono-atomic steps that are always present on all catalytic surfaces of practical use. From low-energy electron microscopy and atomic force microscopy data, we are able to set up an extended Wulff construction scheme and provide a clear elaboration of different interactions governing the equilibrium shapes of the growing hBN islands that deviate from the idealistic triangular form. Most importantly, intrinsic hBN edge energy and interaction with the iridium step edges are examined separately, revealing in such way the importance of substrate step morphology for the island structure and the overall quality of 2D materials.

## Introduction

Production of single- and multi-layer hexagonal boron nitride (hBN) samples with minimum amount of defects has developed into one of the most important areas of investigation of this insulating 2D material (2DM) exhibiting high chemical stability and excellent thermal conductivity^1–3^. Elimination of defects from the production process is essential for scalable, high-throughput synthesis of hBN that holds a great potential for advancements in various fields of technology, such as field effect transistors^4^, light-emitting diodes^5^ and sensors^6^. The method enabling such synthesis is chemical vapor deposition (CVD), which in the case of hBN typically consists of initial nucleation of individual islands on a catalyst metal surface, followed by island growth and coalescence to form a full monolayer^7^. When neighboring islands merge, defects are formed at the boundary, resulting in lower material quality and deterioration of device performance^8,9^. Since island coalescence is an unavoidable step in CVD synthesis, it is important to understand all aspects of island nucleation, shape, and growth, in order to develop new routes for synthesis optimization.

Single-layer hBN has been grown via CVD on a wide range of metal substrates, e.g., on Ru, Rh, Ni, Ir, Pd, Pt, Cu and Fe^7,10–18^. Initially, hBN islands are often zig-zag (ZZ) terminated triangles, with possible exceptions for some growth conditions^19–21^. The triangles exhibit two dominant orientations, which originate from the bi-elemental hBN unit cell^12^. Further evolution of island shape, and therefore the domain boundaries later on, can be altered during CVD by adjusting the accessible parameters (e.g., temperature or precursor pressure/flux^19^), but the choice of a particular substrate with its specific morphology is a crucial initial factor, since precursor-substrate and hBN-substrate interactions dictate the course of the synthesis.

A very important feature of substrate morphology are surface steps. They are always present on both single-crystalline and poly-crystalline foil substrates, and are often sites of hBN nucleation^22–24^. Also, island growth anisotropy induced by the substrate steps has been observed for hBN on Ru(0001)^25^, Pt(111)^26^, Ir(111)^23^, and Cu(110)^27^, and in a similar manner for graphene on metals^22,28,29^. Due to the increased binding of the edge of 2DMs to the substrate step edge, step-up (and in some cases also step-down) expansion is hindered and causes anisotropic growth rates of the islands. However, up to now very little attention has been devoted to disentangling the different energy factors that contribute to the observed island shapes. Here, we elucidate the origin of hBN island shape anisotropy on metals by performing a case study of hBN growth on Ir(111) with low-energy electron microscopy (LEEM). We explicitly consider the intrinsic energy of hBN island edges^30^, the islands’ binding to the substrate, and the specifics of the interaction between hBN islands and substrate step edges. Such detailed study is feasible because hBN-Ir interaction allows hBN growth over the Ir step edges while leaving a clear mark on the islands’ shape in the form of distinct triangles and trapezoids^23^.

## Results and Discussion

**Figure 1:**
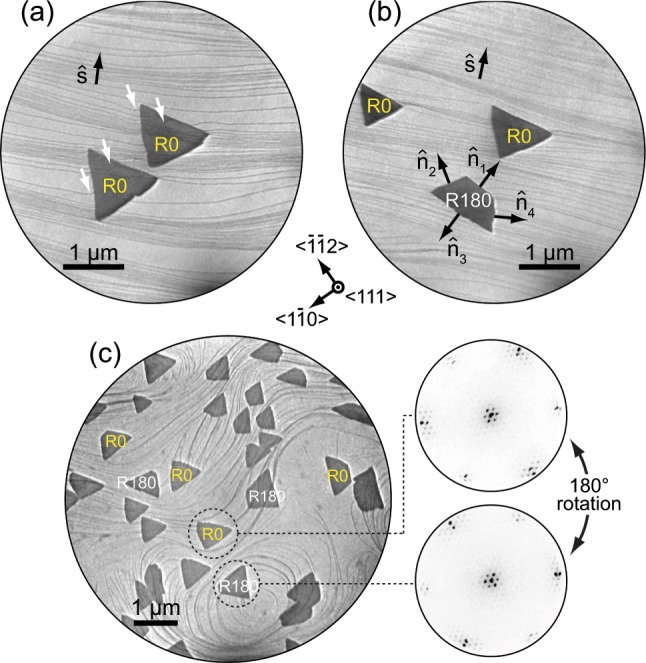
Shape and orientation of hBN islands. (**a**,**b**) LEEM images of hBN islands on Ir(111) with uniform $\hat{s}$ (designating Ir step-up direction). White arrows indicate sites of strong Ir step edge bending. Edge normals $\hat{n}$_i_ are noted for one of the islands in panel (**b**). (**c**) LEEM image of R0 and R180 islands, including two representative *μ*-LEED patterns, on a part of the Ir surface with strongly varying $\hat{s}$. (**a**,**b**) *E* = 24.1 eV, (**c**) *E* = 17.5 eV in LEEM and *E* = 35.2 eV in *μ*-LEED. Crystallographic directions noted in the center of the figure apply to all LEEM and *μ*-LEED images.

LEEM images in Fig. 1(a,b) show isolated hBN islands on the Ir surface, where thin dark lines spanning across the field of view are Ir step edges. Crystallographic analysis, taking into account image rotation in LEEM (e.g., see ref. ^21^ for technical details), reveals that the hBN edges are of ZZ type. The orientation of hBN island edges can be described by unit vectors $\hat{n}$ which are perpendicular to the edges, as shown in Fig. 1(b). The local orientation of Ir steps is designated by a unit vector $\hat{s}$ which for every point along the step is perpendicular to the step. The orientation of $\hat{s}$, i.e., the difference between step-up and step-down direction, can be determined by recognizing that the short base of the trapezoid must be facing the Ir step-up direction [see atomic force microscope (AFM) data below]. When straight steps are present on the Ir surface and $\hat{s}$ shows minor change across a large area, as in Fig. 1(a,b), one hBN orientation (denoted as R0) grows exclusively in triangular form, and the other one (rotated by 180°, denoted as R180) grows exclusively in the form of trapezoids, as we reported earlier^23^ (see also Supplementary Section S1 for the LEEM image covering a larger field of view). The shape of islands changes, however, when the Ir substrate exhibits a complex surface morphology and contains step edges with large curvature, including hills and valleys. Such a situation is shown in Fig. 1(c), where many R0 and R180 islands are visible (in this particular case, the borazine pressure during synthesis was 6 × 10^−8^ mbar, leading to a higher island density). Whether the islands are of R0 or R180 orientation can be deduced (i) by simply measuring the orientation of their edges, or (ii) by comparing the islands’ 3-fold symmetric selected-area low-energy electron (*μ*-LEED) patterns as illustrated in Fig. 1(c).

A careful inspection of LEEM images reveals that R0 islands are triangular on some parts of the surface, while they are trapezoidal on other parts [see labeled islands in Fig. 1(c)]. Moreover, the short base of trapezoidal islands is found at different positions, i.e., the triangles truncation occurs at different vertices in order to form trapezoids. This is also valid for R180 islands. Considering that the short base of trapezoidal islands faces the step-up direction of Ir, we deduce that R0 and R180 islands are not predetermined to grow as triangles or trapezoids, but their shape is governed by Ir steps. Complex substrate step morphology, as in Fig. 1(c), also allows for the formation of irregular hBN islands, i.e., islands with interior angles other than 60° and 120°, and their origin will be discussed later in the text.

**Figure 2:**
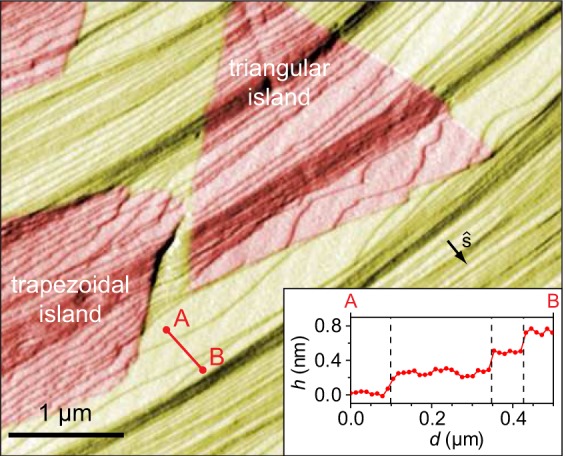
AFM image (first derivative of topography in *x*) of hBN islands (red) on Ir(111) (yellow). Thin diagonal lines are Ir step edges. The inset shows line profile between points *A* and *B* prior to AFM image differentiation, vertical dashed lines mark positions of Ir step edges.

Close to the hBN edges, Ir steps are often strongly bent, as marked by white arrows in Fig. 1(a), indicating that the interaction between hBN island edges and Ir step edges is significant and plays an important role in the growth of hBN. Recently, Poelsema *et al*. also found that the growth of hBN on Ir(111) results in a severe reorientation of Ir step edges^31^. A better view of step layout can be obtained from scanning probe imaging. In Fig. 2, an AFM image with several hBN islands is shown. The step-up direction is easily identified from an AFM profile shown in the inset. Ir step edges, which are rather straight in the hBN-free region, are distorted in areas where hBN islands overgrew them. This is most prominent at the lower right edge of the triangular island that faces the Ir step-up direction, where Ir steps exhibit a wavy structure and contain straight segments parallel to the hBN edge. Furthermore, in Fig. 2, a short base of the trapezoidal island has formed in the step-up direction, facilitating in such way parallel configuration of hBN edge and Ir steps. Our AFM data suggests that strong hBN-Ir interaction favors attachment of hBN edges to Ir step edges which is enabled by their parallel alignment, most prominently in the step-up direction of Ir.

A theoretical study by Bets *et al*. also showed that docking of hBN edges to the surface steps of metal substrates is energetically beneficial^32^. There, the authors find that such docking, with the assumption of rigid, immobile substrate steps, can result in hBN island shapes that differ form regular, equilateral triangles. However, their results are not directly applicable to the system studied here, since the Ir steps undergo reconfiguration during hBN growth, and hBN islands are able to preserve regular shapes (with interior angles of 60° and 120°) while being attached to the Ir steps at the same time. Evidently, our case scenario is possible due to the strong binding of ZZ edges of hBN to the dense-packed atomic rows of Ir, which originates from the charge redistribution among B, N and Ir atoms^24,33^. Such strong binding ensures locking of hBN orientation to either R0 or R180 in the early stages of growth^34,35^, and also causes evanescence of an unfavorable polarity of unbound ZZ edges and the associated charge disbalance of hBN islands^20^.

**Figure 3:**
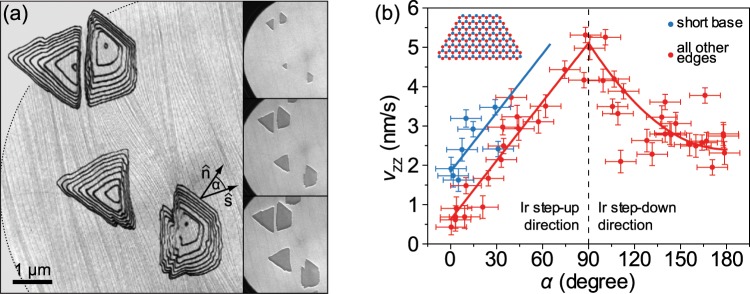
Evolution of hBN islands’ shape. (**a**) Stacked contours of hBN islands extracted from a LEEM growth sequence, overlaid on top of a LEEM image showing clean Ir surface prior to hBN growth. Contours correspond to islands perimeters recorded at time intervals of 32 s. Edge normal $\hat{n}$, local step-up direction $\hat{s}$ and an angle between them *α* are noted for one hBN edge. Three characteristic images of hBN growth are shown in the panels on the right. *E* = 17.3 eV. (**b**) Data plot of ZZ edge advancement speed *v*_ZZ_ as a function of angle *α*. Red and blue lines are the fits to the data.

We now proceed to discuss the evolution of the island shape during growth. Contours of several hBN islands extracted from LEEM data are shown in Fig. 3(a), corresponding to the ZZ-type perimeters of R0 and R180 islands recorded at time intervals of 32 s. The island growth is quantified by measuring edge distances from the island nucleation site, *d*, and calculating the average advancement speed as *v*_ZZ_ = *d*/*t*. At the same time, the angle *α* between $\hat{n}$ and $\hat{s}$ has been measured for each edge, thus providing information to plot *v*_ZZ_ as a function of *α*. In total, 16 islands have been analyzed, and from the data shown in Fig. 3(b) it is clear that hBN edges propagate faster (slower) when their normals $\hat{n}$ are perpendicular (parallel) to the local direction of $\hat{s}$. Short bases of trapezoids (blue dots) have been singled out from all other edges (red dots) because of their different elemental composition (see inset). The data in Fig. 3(b) shows functional dependence, and we fit it with simple mathematical functions in order to establish an analytical model of hBN growth. At *α* = 90°, the island edge changes its growth orientation from step-up to step-down with respect to the Ir surface, and it is reasonable to assume modification of hBN-Ir interaction and also a different behavior of *v*_ZZ_(*α*). Therefore, we fit the data with a combination of linear (for *α* ≤ 90°) and quadratic (for *α* > 90°) functions (see Supplementary Section S2 for fit details).

From a comparison of the product *v*_ZZ_*L*, where *L* is the typical island size, with the values of the diffusion coefficient *D* of various borazine fragments, i.e., the building blocks for hBN growth, it is clear that *D* ≫ *v*_ZZ_*L* (see Supplementary Section S3 for more details). This implies that hBN growth on Ir(111) is taking place near the thermodynamic equilibrium and that *v*_ZZ_ is proportional to the edge free energy^36^. Likewise, any changes of the edge binding energy are reflected in the modification of *v*_ZZ_, which will be very important in the subsequent analysis. Since our system is close to the thermodynamic equilibrium, the thermodynamic Wulff construction can be applied to obtain the shape of 2D islands^36,37^, and hence we use it to reconstruct the observed R0 and R180 island forms. We use an analytic expression, originally derived to describe graphene edges^38^, for the epitaxial hBN island edge energy per unit length as a function of polar angle and chemical potential difference, *γ*(*χ*, Δ*μ*)^30,37^ (see Supplementary Section S4 for details). The chemical potential Δ*μ* is defined as a disbalance between chemical potentials of B and N atoms, Δ*μ* = (*μ*_B_ − *μ*_N_)/2. Highly positive Δ*μ* favors B-terminated ZZ edges, while highly negative Δ*μ* favors N-terminated ZZ edges. *γ*(*χ*, Δ*μ*) also contains binding energies of ZZ and armchair edges to Ir that can be found in the literature^24,33,39^.

**Figure 4:**
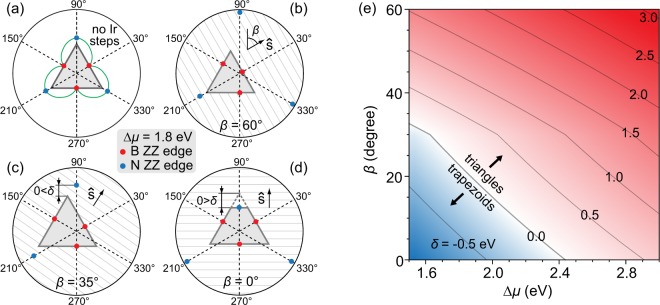
Formation of a trapezoidal hBN island at different growth conditions. (**a**) Thermodynamic Wulff construction of an hBN island on Ir without steps. Green line is *γ*(*χ*, Δ*μ*), and gray triangle indicates the Wulff shape. (**b**–**d**) Transition of a triangular island shape to trapezoidal after including the interaction of hBN with the Ir steps and subsequent rotation of $\hat{s}$. Gray lines indicate Ir steps. (**e**) Plot of *δ*(*β*, Δ*μ*). For *δ* < 0 (blue region) and *δ* > 0 (red region), trapezoidal and triangular islands grow on the Ir surface, respectively.

To be consistent with the realistic experimental values of Δ*μ*^33^, and also with the preference of B-terminated ZZ edges^23,24^, our analysis is restricted to 1.5 eV < Δ*μ* < 3 eV. A comparable analysis with N-terminated island edges, where Δ*μ* < 0, is straightforward. The Wulff construction of an hBN island at Δ*μ* = 1.8 eV is shown in the polar plot in Fig. 4(a). The edge energy *γ*(*χ*, Δ*μ*) used in this construction includes the hBN island’s intrinsic edge energy and the binding of hBN to the flat Ir substrate without steps (details are given in the Supplementary Section S4). The island shape is determined by the red points of *γ*(*χ*, Δ*μ*) which correspond to B-terminated ZZ edges. N-terminated ZZ edges, designated by blue points, have higher energy and therefore do not constitute the edges of hBN island at these conditions. It follows from Fig. 3 and the outlined diffusion considerations that *v*_ZZ_(*α*) corresponds to the ZZ edge energy modulation arising from the relative edge orientation with respect to the Ir steps. This is incorporated into the Wulff construction by applying *γ*(*χ*, Δ*μ*) → *γ*(*χ*, Δ*μ*) · *v*_ZZ_(*χ*) and by repositioning the red and the blue points in Fig. 4(b–d) accordingly. Our LEEM data shows that these few points are the only relevant ones to describe the shape of hBN islands.

For the sake of clarity, we focus on the top vertex and the upper-right edge of the triangular hBN island in Fig. 4(a–d) to examine the truncation effect of the island. Introduction of Ir steps modifies the energies of all hBN edges, depending on the orientation of hBN island with respect to Ir steps. This orientation is quantified by the angle *β* measured between $\hat{s}$ and direction corresponding to *χ* = 90° [see Fig. 4(b)]. For relatively large values of *β* as in Fig. 4(b,c), Ir step-bending as visible in the AFM data of Fig. 2 is the optimal mechanism for hBN island energy minimization. The required bending at the upper-right island edge is not large (step energy increases with the step curvature^40^) and its cost is compensated by an overall energy gain achieved by strong binding between parallel hBN edge and Ir steps. In such a situation, B-terminated ZZ edges remain energetically preferred. As *β* decreases, the cost of Ir step bending becomes too high and it becomes energetically non-profitable. However, the energy of N-terminated ZZ edge at the top of the island in Fig. 4(d) is reduced significantly since it becomes (nearly) parallel with Ir steps, resulting in hBN island truncation at the vertex pointing in the step-up direction. After truncation, at the short base of the trapezoidal island, it takes much less (if any) Ir step bending to achieve a parallel configuration and strong binding between hBN island edge and Ir steps, and in such way an initially unfavorable N-termination of hBN islands is energetically compensated.

The presented Wulff construction predicts, in agreement with our LEEM data, that the truncation is allowed only in the Ir step-up direction. It can be argued that hBN edges are bound to the metal atoms more strongly in the step-up direction as compared to the step-down direction due to the anisotropic passivization, similarly to the case of epitaxial graphene^29^. Binding of different hBN edges to Ir steps that undergo bending and repositioning constitutes the energetic background of *v*_ZZ_(*α*). That is why the inclusion of *v*_ZZ_(*α*) into the Wulff construction is crucial for obtaining the experimentally observed shapes of hBN islands. We note that hBN edges other than ZZ have been found in our experiments. Such edges become energetically stable when hBN islands form on an Ir region with significant step curvature [see Fig. 1(c)], where the angle *α* changes as a function of time and position along a particular hBN edge. Our analysis could be further refined so that it would include such non-ZZ edges on a complex substrate step morphology, but that would surpass the scope of the present study.

A systematic investigation of the truncation effect is shown in Fig. 4(e) in which *δ*, i.e., the vertical separation between N-terminated ZZ edge and its closest vertex as depicted in Fig. 4(c,d), is plotted as a function of Δ*μ* and *β*. This allows for examination and prediction of hBN island shapes at various growth conditions (different temperatures or precursor pressures, leading to the modification of Δ*μ*) and different substrate step morphologies. For certain (*β*, Δ*μ*) combinations, trapezoidal islands (*δ* < 0, blue region) are energetically preferred over triangular ones (*δ* > 0, red region). This explains why R0 and R180 islands have different shapes on the surface with uniform $\hat{s}$, and also why do they change their shape when $\hat{s}$ (i.e., *β*) changes. The level of truncation of the triangle also depends on Δ*μ* and ***β***, explaining trapezoids of different heights in Fig. 1(c). In N-rich growth conditions (at lower Δ*μ* values), trapezoidal islands are more likely to be found on the Ir surface since the triangle truncation becomes preferable for a wider range of Ir step orientations. In a B-rich growth environment (at higher Δ*μ* values), all hBN edges will be B-terminated irrespective of the angle *β*, and only triangular islands would be present on the Ir surface for any orientation of Ir steps.

## Conclusions

We have shown that the step morphology of the substrate used in CVD growth of hBN is a crucial factor which determines the energetically most stable shape of synthesized hBN islands. The total energy of the system is minimized by adhering hBN edges to the Ir step edges, which is achieved by repositioning of Ir steps and formation of trapezoids (instead of triangles) during hBN growth. The degree of Ir step repositioning and the feasibility of trapezoidal shape depend on the relative orientation between hBN island edges and Ir steps, and the chemical potentials of B and N atoms during the synthesis of hBN. The use of an extended Wul construction allowed exact pinpointing of di erent energy contributions governing hBN growth, and this enables the application of our results, by adjusting the relevant interaction parameters, in studies of hBN synthesis on other metal substrates.

## Methods

Single-layer hBN was grown on Ir(111) in an ultra-high vacuum setup via CVD by using borazine (Katchem) as a precursor. The Ir single-crystal (Mateck) was cleaned by Ar sputtering at 2 keV followed by heating in oxygen at 1170 K and annealing at 1470 K. Unless otherwise noted, the borazine pressure during CVD was 10^−8^ mbar and the temperature was 1170 K. An Elmitec SPE-LEEM III microscope was used to carry out *in-situ*, bright field LEEM and *μ*-LEED measurements. AFM measurements were performed *ex-situ* in air with a Veeco Dimension 3100 microscope operated in tapping mode.

## Supplementary information

Supplementary Information

## Data Availability

All data needed to evaluate the conclusions in the paper are present in the paper and/or the Supplementary Information. Additional data related to this paper may be requested from the corresponding author.

## References

[1] Nagashima A, Tejima N, Gamou Y, Kawai T, Oshima C (1995). Electronic Structure of Monolayer Hexagonal Boron Nitride Physisorbed on Metal Surfaces. Phys. Rev. Lett..

[2] Pakdel A, Bando Y, Golberg D (2014). Nano boron nitride flatland. Chem. Soc. Rev..

[3] Zhang K, Feng Y, Wang F, Yang Z, Wang J (2017). Two dimensional hexagonal boron nitride (2D-hBN): synthesis, properties and applications. J. Mater. Chem. C.

[4] Roy T (2014). Field-Effect Transistors Built from All Two-Dimensional Material Components. ACS Nano.

[5] Ross JS (2014). Electrically tunable excitonic light-emitting diodes based on monolayer WSe2 p–n junctions. Nat. Nanotechnol..

[6] Sajjad M, Morell G, Feng P (2013). Advance in Novel Boron Nitride Nanosheets to Nanoelectronic Device Applications. ACS Appl. Mater. Interfaces.

[7] Kim KK (2012). Synthesis of Monolayer Hexagonal Boron Nitride on Cu Foil Using Chemical Vapor Deposition. Nano Lett..

[8] Gibb AL (2013). Atomic Resolution Imaging of Grain Boundary Defects in Monolayer Chemical Vapor Deposition-Grown Hexagonal Boron Nitride. J. Am. Chem. Soc..

[9] Li Q (2015). Grain Boundary Structures and Electronic Properties of Hexagonal Boron Nitride on Cu(111). Nano Lett..

[10] Goriachko A (2007). Self-Assembly of a Hexagonal Boron Nitride Nanomesh on Ru(0001). Langmuir.

[11] Corso M (2004). Boron nitride nanomesh. Science.

[12] Auwärter W, Muntwiler M, Osterwalder J, Greber T (2003). Defect lines and two-domain structure of hexagonal boron nitride films on Ni(111). Surf. Sci..

[13] Lee Y-H (2012). Growth selectivity of hexagonal-boron nitride layers on Ni with various crystal orientations. RSC Adv..

[14] Orlando F (2012). Epitaxial Growth of Hexagonal Boron Nitride on Ir(111). J. Phys. Chem. C.

[15] Morscher M, Corso M, Greber T, Osterwalder J (2006). Formation of single layer h-BN on Pd(111). Surf. Sci..

[16] Ćavar E (2008). A single h-BN layer on Pt(111). Surf. Sci..

[17] Joshi S (2012). Boron Nitride on Cu(111): An Electronically Corrugated Monolayer. Nano Lett..

[18] Vinogradov NA (2012). One-Dimensional Corrugation of the h -BN Monolayer on Fe(110). Langmuir.

[19] Stehle Y (2015). Synthesis of Hexagonal Boron Nitride Monolayer: Control of Nucleation and Crystal Morphology. Chem. Mater..

[20] Poelsema B (2019). Polar edges and their consequences for the structure and shape of hBN islands. 2D Mater..

[21] Felter J, Raths M, Franke M, Kumpf C (2019). *In situ* study of two-dimensional dendritic growth of hexagonal boron nitride. 2D Mater..

[22] Sutter PW, Flege J-I, Sutter EA (2008). Epitaxial graphene on ruthenium. Nat. Mater..

[23] Petrović M, Hagemann U, Horn-von Hoegen M, Meyer zu Heringdorf F-J (2017). Microanalysis of single-layer hexagonal boron nitride islands on Ir(111). Appl. Surf. Sci..

[24] Farwick zum Hagen FH (2016). Structure and Growth of Hexagonal Boron Nitride on Ir(111). ACS Nano.

[25] Sutter P, Lahiri J, Albrecht P, Sutter E (2011). Chemical Vapor Deposition and Etching of High-Quality Monolayer Hexagonal Boron Nitride Films. ACS Nano.

[26] Zhang Y (2015). Hexagonal Boron Nitride Cover on Pt(111): A New Route to Tune Molecule– Metal Interaction and Metal-Catalyzed Reactions. Nano Lett..

[27] Wang L (2019). Epitaxial growth of a 100-square-centimetre single-crystal hexagonal boron nitride monolayer on copper. Nature.

[28] Loginova E, Bartelt NC, Feibelman PJ, McCarty KF (2009). Factors influencing graphene growth on metal surfaces. New J. Phys..

[29] Wang Z-J (2016). Stacking sequence and interlayer coupling in few-layer graphene revealed by *in situ* imaging. Nat. Commun..

[30] Liu Y, Bhowmick S, Yakobson BI (2011). BN White Graphene with “ Colorful” Edges: The Energies and Morphology. Nano Lett..

[31] Poelsema B, Zandvliet HJW, van Houselt A (2019). Stoichiometric edges during the intrinsic growth of hexagonal boron nitride on Ir(111). New J. Phys..

[32] Bets KV, Gupta N, Yakobson BI (2019). How the Complementarity at Vicinal Steps Enables Growth of 2D Monocrystals. Nano Lett..

[33] Zhao R, Li F, Liu Z, Liu Z, Ding F (2015). The transition metal surface passivated edges of hexagonal boron nitride (h-BN) and the mechanism of h-BN’ s chemical vapor deposition (CVD) growth. Phys. Chem. Chem. Phys..

[34] Camilli L, Sutter E, Sutter P (2014). Growth of two-dimensional materials on non-catalytic substrates: h-BN/Au(111). 2D Mater..

[35] Song X (2015). Chemical vapor deposition growth of large-scale hexagonal boron nitride with controllable orientation. Nano Res..

[36] Artyukhov VI, Liu Y, Yakobson BI (2012). Equilibrium at the edge and atomistic mechanisms of graphene growth. Proc. Natl. Acad. Sci. USA.

[37] Zhang Z, Liu Y, Yang Y, Yakobson BI (2016). Growth Mechanism and Morphology of Hexagonal Boron Nitride. Nano Lett..

[38] Liu Y, Dobrinsky A, Yakobson BI (2010). Graphene Edge from Armchair to Zigzag: The Origins of Nanotube Chirality?. Phys. Rev. Lett..

[39] Laskowski R, Blaha P, Schwarz K (2008). Bonding of hexagonal BN to transition metal surfaces: An ab initio density-functional theory study. Phys. Rev. B.

[40] Giesen M (2001). Step and island dynamics at solid/vacuum and solid/liquid interfaces. Prog. Surf. Sci..

