# Supplementary material for: Equilibrium shape of single-layer hexagonal boron nitride islands on iridium
Source: Sci Rep. 2019 Dec 20;9:19553. doi: 10.1038/s41598-019-56000-1 (PMC6925269; doi:10.1038/s41598-019-56000-1)
Supplement: Supplementary file 1 — Supplementary Information [file 41598_2019_56000_MOESM1_ESM.pdf]

SUPPLEMENTARY INFORMATION

Equilibrium shape of single-layer hexagonal boron nitride islands on iridium

Marin Petrović,[1,2,a)] Michael Horn-von Hoegen,[1] and Frank J. Meyer zu Heringdorf[1]

[1)] *Faculty of Physics and CENIDE, University of Duisburg-Essen, Lotharstr. 1, D-47057 Duisburg, Germany*

[2)] *Center of Excellence for Advanced Materials and Sensing Devices, Institute of Physics, Bijenička cesta 46, HR-10000 Zagreb, Croatia*

[a)] Electronic mail: mpetrovic@ifs.hr

S1. OVERVIEW OF HBN ISLANDS SHAPES IN A LARGE FIELD OF VIEW

Fig. S1 shows a large area (25 µm in diameter) of the Ir(111) substrate with many hBN islands present on the surface. All islands can be categorized as triangular or trapezoidal in shape, with possible deviations from the ideal, regular forms having interior angles of 60° and 120°.

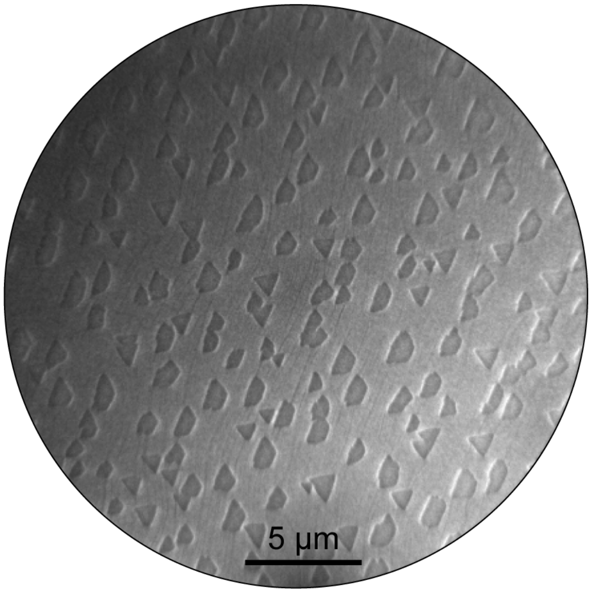

Figure S1. LEEM image of a large number of triangular and trapezoidal hBN islands on Ir(111).

S2. DETAILS OF THE $v_{\mathrm{ZZ}}(\alpha)$ DATA FIT

The red data points in Fig. 3(b), corresponding to B-terminated ZZ edges of hBN, were fitted with a combination of linear and quadratic functions

$$v_{\mathrm{ZZ,B}}(\alpha) = \begin{cases} c_1 + c_2\alpha, & 0° \leq \alpha \leq 90° \\ c_3 + c_4\alpha + c_5\alpha^2, & 90° \leq \alpha \leq 180° \end{cases}$$

and the blue data points corresponding to N-terminated ZZ edges of hBN were fitted with a linear function

$$v_{\mathrm{ZZ,N}}(\alpha) = c_1^{'} + c_2\alpha$$

having the same slope as the one fitting the red data points, only with different intercept. The application of a particular fit model (linear or quadratic) to the two regions does not qualitatively affect the rest of the analysis nor it brings any novelties. The choice of the fitting functions as shown above provided a good fit to the data points (the adjusted R-Square value of 0.90 for $0° \leq \alpha \leq 90°$ and 0.42 for $90° \leq \alpha \leq 180°$) and was therefore used further in our study. After requiring continuity at $\alpha = 90°$ and a minimum at $\alpha = 180°$, the fit parameters for v_{ZZ} in nm/s and α in degrees are given in Table S1.

**parameter**	**value**
c_1	0.6 ± 0.2
c_2	0.050 ± 0.004
c_3	13.2 ± 0.6
c_4	-0.120 ± 0.007
c_5	$(3.3 \pm 0.2) \times 10^{-4}$
$c_1^{'}$	1.8 ± 0.2

Table S1. Parameters of the $v_{ZZ}(\alpha)$ fitting functions.

Error bars in Fig. 3(b) have been added to account for deviation of $\hat{\boldsymbol{s}}$ and $\hat{\boldsymbol{n}}$ as the islands grow, finite pixel size and smearing of hBN island edges in LEEM images, and deceleration effects related to the vicinity of neighboring islands.

S3. EVALUATION OF THE DIFFUSION COEFFICIENTS

The surface diffusion coefficient D for an energy barrier E at a temperature T can be calculated with the aid of equation $D = (k_B T n l^2/4h) \exp(-E/k_B T)$[S1], where k_B is the Boltzmann constant, h is the Planck constant, n is the number of neighboring sites available for diffusion that are separated by distance l [$n = 3$ and $l = 1.568$ Å for Ir(111)[S2]]. By utilizing the typical energies for surface diffusion barriers of B atoms, N atoms and BN dimers on transition metals (all in the range 0.1 - 1 eV[S3]), one obtains D larger by several orders of magnitude than the product $v_{ZZ}L \approx 3\text{nm/s} \cdot 1\mu\text{m}$. Therefore, in our experiments hBN islands grow near thermodynamic equilibrium, meaning that the edge advancement speed is directly proportional to the edge free energy and that thermodynamic Wulff construction can be used for the construction of the island shapes[S4].

S4. DETAILS OF HBN EDGE ENERGY

An analytic expression for epitaxial hBN island edge energy per unit length as a function of polar angle and chemical potential has the form[S3,S5,S6] $\gamma(\chi, \Delta\mu) = |\gamma_0| \cos(\chi + C)$, where $|\gamma_0| = 2\left(\gamma_{\mathrm{A}}^2 + \gamma_{\mathrm{Zx}}^2 - \sqrt{3}\gamma_{\mathrm{A}}\gamma_{\mathrm{Zx}}\right)^{1/2}$, $C = \operatorname{sgn}(\chi) \cdot \arctan\left(\sqrt{3} - 2\gamma_{\mathrm{Zx}}/\gamma_{\mathrm{A}}\right)$, and the subscript x is "N" for $-30° < \chi < 0°$ and it is "B" for $0° < \chi < 30°$. The intrinsic energies of B- and N-terminated zig-zag (γ_{ZB} and γ_{ZN}) and armchair (γ_{A}) edges with the inclusion of binding to the flat metal substrate are

$$\begin{aligned} \gamma_{\mathrm{ZB}} &= 3.3 - \Delta\mu/3 - E_{\mathrm{b,ZB}} \\ \gamma_{\mathrm{ZN}} &= 2.7 + \Delta\mu/3 - E_{\mathrm{b,ZN}} \\ \gamma_{\mathrm{A}} &= 1.9 - E_{\mathrm{b,A}} \end{aligned} \tag{S1}$$

where the binding energy of the ZZ edges to the flat Ir is $E_{\mathrm{b,ZB}} = 2.0$ eV and $E_{\mathrm{b,ZN}} = 1.6$ eV per unit cell, as obtained from the DFT calculations[S7]. The value of $E_{\mathrm{b,A}}$, i.e. the binding energy of the armchair edge to the flat Ir, was calculated by assuming that the ratio of binding energies of extended hBN sheets to Ir(111) and Rh(111)[S8] (the two elements of the same group) is the same as the ratio of hBN armchair edge binding energies to the same substrates[S9], yielding $E_{\mathrm{b,A}} = 0.59$ eV per unit cell. The chemical potential $\Delta\mu$ is defined as a disbalance between chemical potentials of B and N atoms, $\Delta\mu = (\mu_{\mathrm{B}} - \mu_{\mathrm{N}})/2$.

It is important to note that the equations outlined up to now do not include interaction with the Ir steps, and can therefore be used for construction of equilibrium shape of hBN island on a flat (step-less) Ir substrate only, as shown in Fig. 4(a). hBN-Ir step interaction is included by making the substitution $\gamma(\chi, \Delta\mu) \to \gamma(\chi, \Delta\mu) \cdot v_{\mathrm{ZZ}}(\chi)$, resulting in an alteration of the hBN island shape, as depicted in Figs. 4(b)-(d). Such substitution is justified since $v_{\mathrm{ZZ}}(\alpha)$ provides direct information about the relative modification of the energy of different ZZ edges, and only these relative (not absolute) changes are needed for reconstruction of the experimental hBN forms.

N-terminated ZZ edges growing in the step-down direction of Ir were never found in our experiments, and blue data points do not exist for $90° < \alpha < 180°$ in the $v_{\mathrm{ZZ}}(\alpha)$ plot in Fig. 3(b). In order to estimate $\gamma(\chi, \Delta\mu) \cdot v_{\mathrm{ZZ}}(\chi)$ for N-terminated ZZ edges in the entire range of angles, which is required for plotting all of the blue points in Figs. 4(b)-(d), we assume that the offset between $v_{\mathrm{ZZ,B}}(\alpha)$ and $v_{\mathrm{ZZ,N}}(\alpha)$ is the same for $0° < \alpha < 90°$ and

$90° < \alpha < 180°$, and is equal to $c_1^{'} - c_1$.

REFERENCES

[S1] J. Renard, M. B. Lundeberg, J. A. Folk, and Y. Pennec, Phys. Rev. Lett. **106**, 156101 (2011).

[S2] J. W. Arblaster, Platinum Met. Rev. **54**, 93 (2010).

[S3] Z. Zhang, Y. Liu, Y. Yang, and B. I. Yakobson, Nano Lett. **16**, 1398 (2016).

[S4] V. I. Artyukhov, Y. Liu, and B. I. Yakobson, Proc. Natl. Acad. Sci. U.S.A. **109**, 15136 (2012).

[S5] Y. Liu, A. Dobrinsky, and B. I. Yakobson, Physical Review Letters **105**, 235502 (2010).

[S6] Y. Liu, S. Bhowmick, and B. I. Yakobson, Nano Lett. **11**, 3113 (2011).

[S7] F. H. Farwick zum Hagen, D. M. Zimmermann, C. C. Silva, C. Schlueter, N. Atodiresei, W. Jolie, A. J. Martínez-Galera, D. Dombrowski, U. A. Schröder, M. Will, P. Lazić, V. Caciuc, S. Blügel, T.-L. Lee, T. Michely, and C. Busse, ACS Nano **10**, 11012 (2016).

[S8] R. Laskowski, P. Blaha, and K. Schwarz, Phys. Rev. B **78**, 045409 (2008).

[S9] R. Zhao, F. Li, Z. Liu, Z. Liu, and F. Ding, Phys. Chem. Chem. Phys. **17**, 29327 (2015).
